# Differences in Epidemiology and Risk Factors for Atrial Fibrillation Between Women and Men

**DOI:** 10.3389/fcvm.2020.00003

**Published:** 2020-01-31

**Authors:** Maryam Kavousi

**Affiliations:** Department of Epidemiology, Erasmus MC, University Medical Center Rotterdam, Rotterdam, Netherlands

**Keywords:** atrial fibrillation, sex, gender, epidemiology, incidence, prevalence, risk factors

## Abstract

Atrial fibrillation (AF), the most common sustained cardiac arrhythmia, is one of the most frequent cardiovascular diseases among both women and men. Although age-adjusted AF incidence and prevalence is larger among men, women are older at the time of AF diagnosis and have larger risk for AF-associated adverse outcomes such as morality and stroke. Based on evidence from epidemiological studies, elevated body mass index seems to confer a higher risk of AF among men. However, evidence regarding sex differences in the association between diabetes mellitus, elevated blood pressure, and dysglycemia with AF remains conflicting. While men with AF have larger burden of coronary artery disease, women with AF tend to have a larger prevalence of heart failure and valvular heart disease. Recently, several women-specific risk factors including pregnancy and its complications and number of children have been associated with AF. Earlier age at menopause, despite being a strong marker of adverse cardiometabolic risk, does not seem to be associated with increased risk of AF. To reduce the AF burden in both genders, better understanding of the differences between women and men with regard to AF is central. Large-scale studies are needed to separately investigate and report on women and men. Besides observations from epidemiological and clinical studies, to improve our understanding of sexual dimorphism in AF, sufficiently large genome-wide association studies as well as well-powered Mendelian randomization studies are essential to shed light on the sex-specific nature of the associations of risk factors with AF.

## Introduction

Atrial fibrillation (AF), the most common sustained cardiac arrhythmia, is one of the most frequent cardiovascular diseases (CVD) among both women and men. Verification and diagnosis of AF is based on the electrocardiogram (ECG). The symptoms secondary to AF are broad. Irregular palpitations, dyspnea on exertion, and sensation of lightheadedness are among the most common complaints. Other, more general, symptoms include fatigue, weakness or generalized malaise, and chest discomfort. AF is currently classified as paroxysmal (i.e., AF terminating spontaneously or with intervention within 7 days of onset), persistent AF (i.e., continuous AF, sustained more than 7 days), long-standing persistent (i.e., AF lasting for more than 12 months in duration), and permanent (i.e., AF cases in which any further attempts to restore and/or maintain sinus rhythm is stopped based on the joint decision of the patient and the clinician) (1–3). Besides traditional risk factors; including larger body mass index (BMI), diabetes mellitus (DM), hypertension, smoking, and dyslipidemia, diseases of the heart including history of heart failure (HF), coronary heart disease (CHD), and valvular heart disease are among the major risk factors for AF. Recently, several women-specific risk factors including pregnancy and its complications and number of children have been associated with AF. Moreover, evidence regarding age of menopause, a strong marker of adverse cardiometabolic risk, in relation to increased risk of AF remains inconclusive. This article provides an overview of the current literature on differences in the epidemiology of AF between women and men including incidence, prevalence, traditional cardiovascular risk factors, and women-specific risk factors. Further, knowledge gaps and areas for future research are highlight.

## Incidence and Prevalence of Atrial Fibrillation

Prevalence AF of in general population increases with advancing age, from 0.12–0.16% in individuals younger than 49 years to 1.7–4.0% in those aged 60–70 years and as high as 13.5–17.8% beyond 80 years of age (4, 5).

The age-adjusted prevalence and incidence of AF is lower among women compared to men (6–8). The Global Burden of Disease (GBD) reported an estimated age-adjusted prevalence (per 1,000 person-years) of 373.1 for women and 596.2 for men which translated to 12.6 million women and 20.9 million men living with AF globally in 2010. The age-adjusted incidence rates (per 1,000 person-years) were 59.5 for women and 77.5 for men globally in 2010. Of note, the GBD reported progressive increases in overall burden, incidence, prevalence, and AF-associated mortality between 1990 and 2010 in both women and men. Although controversial, the mortality associated with AF seems to be higher in women (6).

Epidemiological studies show a high lifetime risk of AF in both women and men (4, 9). At age 55 years, the lifetime risk to develop AF was 22.2% in women and 23.8% in men in the population-based Rotterdam Study (4).

## Risk Factors for Atrial Fibrillation

### Traditional Cardiovascular Risk Factors for Atrial Fibrillation

Evidence from epidemiological studies has shown several traditional cardiovascular risk factors to be associated with AF in both women and men, including elevated BMI, DM, high blood pressure, smoking, and dyslipidemia.

#### Elevated BMI

Larger BMI is an established risk factor for AF among both women and men. Obesity has been shown to increase the risk of developing AF by 49% compared to non-obese individuals (10). In the large BiomarCaRE Consortium comprising almost 80,000 individuals among whom over 4,000 incident AF cases, BMI explained the largest proportion of AF risk (around 20% for overweight and obesity combined) (9). Elevated BMI was reported to account for up to 18% of risk for incident AF among women in the Women's Health Study (WHS) (11). A body of evidence suggests a stronger association between BMI and AF among men, compared with women (9, 12). One standard deviation (SD) increase in BMI was associated with 11% and 14% greater excess risk for AF among men compared to women in the large BiomarCaRE Consortium (9) and in a recent analyses of the prospective Scottish Heart Health Extended Cohort, respectively (12). Among women and men from the Framingham Heart Study (FHS) (13), the Busselton Health Study (14), and the Malmö Diet and Cancer Study (15) larger BMI or obesity was also a stronger risk factor for AF in men than in women, albeit statistically non-significant.

#### Diabetes Mellitus

Although diabetes is a strong risk factor for CVD, its independent association with AF is debated (16). Several (15, 17–20), but not all (21), studies have reported DM as a risk factor for incident AF. A large meta-analysis of 1,686,097 subjects, among whom 108,703 cases of AF, estimated an ~40% greater risk of AF among diabetics compared to non-diabetic participants. The investigators reported a smaller effect estimate for the studies that had adjusted for multiple risk factors compared to age-adjusted only studies. After adjustment for other cardiovascular risk factors, the investigators concluded that the true risk between DM and subsequent risk of AF may be closer to 25% (17). Recently, the BiomarCaRE Consortium and the Scottish Heart Health Extended Cohort did not find an independent association between DM and incident AF (9, 12).

DM seems to remain a significant but modest risk factor for development of AF explaining approximately only 2.5–3% of the burden of AF (15, 17, 22). DM-associated AF risk was found to be most pronounced in young diabetes patients in the Danish nationwide registries (18). Moreover, the DM-associated AF risk seems to be higher with longer duration of treated diabetes and worse glycemic control (23). To date, robust evidence regarding sex differences in the association between DM with AF remains limited. Although in a recent machine learning aided systematic review and meta-analysis, diabetic women were 24% more likely to develop AF than diabetic men (20), other studies have reported no interaction by sex in the association between DM and AF (9, 12, 23).

#### High Blood Pressure

Hypertension is considered as the most important modifiable risk factor for AF (24). Due to its large prevalence in the population, hypertension accounts for more AF cases than other risk factors. In the Atherosclerosis Risk in Communities (ARIC) study, hypertension alone explained about 22% of AF burden (22). In BiomarCaRE Consortium, elevated blood pressure >140 mmHg explained 13.7% and 14.2% of AF burden in men and women, respectively (9). Hypertension exists in 60–80% of patients with established AF (25). The strength of the association between systolic blood pressure and AF is similar among women and men (9, 12, 26). However, hypertension is more prevalent among women with AF compared to men with AF (27).

#### Smoking

Smoking is among the major risk factors for development of AF. The CHARGE-AF consortium reported 1.44 times higher incident of AF among current smokers compared with non-smokers (28). Smoking is associated with AF in a dose-response manner with increasing risk per cigarette-years (29). Although smoking has shown to carry a higher risk for CHD and stroke in women (30, 31), this pattern can not be robustly extended toward AF. Several studies have reported a larger impact of smoking on AF among women (26), while others have found a stronger effect among men (15). Overall, the association between smoking and AF seems to be similar in both sexes (9, 12, 29, 32, 33).

#### Lipids

Existing evidence regarding the association between blood lipids and AF is inconsistent. The ARIC study reported high levels of total and low-density lipoprotein (LDL) cholesterol to be associated with a lower risk of AF, whereas high-density lipoprotein (HDL) cholesterol and triglycerides were not related to AF risk (34). In a meta-analysis of FHS and MESA, low levels of HDL cholesterol and high levels of triglycerides, but not LDL or total cholesterol, were associated with the risk of AF (35). In a large cohort of 28,449 Japanese subjects who underwent annual health examinations, low HDL cholesterol level was associated with an increased risk of developing AF, while high LDL and total cholesterol levels were associated with increased risk of AF (36). These conflicting results point toward existence of “cholesterol paradox” in AF (37). Sex differences in the relationship between lipid profile and AF have not been consistently reported by studies. In the large Japanese annual health examination study, low HDL cholesterol was associated with an increased risk of new-onset AF only in women, but not in men (36). In the BiomarCARE Consortium, total cholesterol was inversely associated with incident AF with a stronger risk reduction in women. The population attributable risk for total cholesterol was 8.6% in women and 3.8% in men (9).

### Cardiac Diseases as Risk Factors for Atrial Fibrillation

#### Heart Failure

HF and AF share common risk factors. Moreover, the two conditions co-exist (26) and predispose to each other (38). In epidemiological studies, presence of HF led to 3.37-fold risk of AF in the Manitoba Follow-Up Study and 2.67-fold risk of AF in the Cardiovascular Health Study (39, 40). Data on differences between women and men regarding the association of HF with AF are scarce. In FHS, HF was associated with 4.5-fold risk of AF in men and 5.9-fold in woman (26). In the RICA HF registry, AF was more common among women with HF compared to men (41). However, in an observational study from the national HF referral center in Vienna, AF was more frequent among men with HF with preserved ejection fraction (HFpEF) compared with women (68.4% in men and 54.7% in women) (42). Of note, the temporal relationship between HF and AF has been investigated in multiple studies since HF can be an outcome of AF.

#### Coronary Heart Disease

The relationship between AF and myocardial infarction (MI) is also bidirectional. CHD has been associated with an increased risk of AF (43), but AF has also been associated with increased risk of MI (44). Women with AF have a lower prevalence of CHD than men with AF (8, 45). Among individuals with AF, SAFETY investigators reported a prevalence of coronary artery disease of 24.8% for women vs. 41.4% for men (45). MI overall is considered to have a stronger association with AF among men. In FHS, MI increased the risk of AF by 40% among men but not women (26). In the recent BiomarCARE Consortium, history of MI increased the risk of AF by 78% in women and by 93% in men. The difference between women and men was not statistically significant. The investigators reported that 3.0% of AF burden in women and 6.1% of AF burden in men was attributed to history of MI (9).

#### Valvular Heart Disease

Women with AF have higher prevalence of valvular heart disease than men with AF (8, 46). In FHS, valvular heart disease was a significantly more potent risk factor for AF in women than in men (odds ratio for the association of vavular heart disease with AF was 3.4 in women vs. 1.8 in men) (26). Consequently, the attributable risk from valvular heart disease was ~3-fold higher in women than men (18% vs. 5%) (26).

### Women-Specific Risk Factors for Atrial Fibrillation

#### Pregnancy

Observational studies suggest that pregnancy, in general, may potentiate supraventricular tachycardias (47). However, prevalence of AF among pregnant women is very rare (48). Older age at pregnancy is a risk factor for AF during pregnancy and AF is more common during the third trimester compared to the first trimester (48). 1.3% of pregnant women with structural heart disease suffer AF or atrial flutter during their pregnancy with a peak at the end of the second trimester. Experiencing AF or flutter during pregnancy among these patients is associated with unfavorable maternal outcome and affects fetal birth weight (49). AF may also occur during the peripartum period mainly due to drugs used for tocolysis (50, 51) or as a manifestation of peripartum cardiomyopathy (52).

#### Hypertensive Disorders of Pregnancy

Among women with preeclampsia, several electrical and electro-mechanical changes have been observed including increase in P wave duration and dispersion and in atrial electro-mechanical coupling interval measured by tissue Doppler (53). These changes are well-known markers for increased AF incidence in normal hearts. It is yet unclear whether these alterations occur, in general, during pregnancy or they happen only during abnormal pregnancy due to the vascular/atrial susceptibility. A recent nested case-control study from Olmsted County, Minnesota, reported women with AF to be more likely to have experienced a hypertensive pregnancy disorder. This relationship was at least partially mediated by associated obesity and hypertension (54).

#### Number of Children

Among 34,639 women from the WHS, women who had four or more pregnancies were 30–50% more likely to develop AF later in life than women with no pregnancies (55). Repeated exposure to physiological, metabolic, or hormonal factors during pregnancy could, at least partly, explain the increased AF risk.

#### Menopause

Earlier age at menopausal is considered a strong marker of adverse cardiometabolic risk and has been associated with increased risk of CVD and cardiovascular mortality. However, epidemiological studies so far have failed to show an association between age at natural menopause and increased risk of AF. The large WHS including over 30,000 women with 1,350 cases of new-onset AF during a median follow-up of 20.5 years, reported no association between earlier age of menopause with risk of AF (compared with age of menopause >54 years as the referent, hazard ratios (HR) for age of menopause: <45 years: 0.82; 45–49 years: 0.90; 50–54 years: 0.89—all statistically non-significant) (56). This is in line with a previous report by the FHS investigators regarding no association between menopausal age with incident AF (HR for age of menopause <45 years: 1.20 and 1.38 vs. age of menopause >53 and 45–53 years, respectively,—both non-significant) (57). Of note, the WHS investigators did not find evidence regarding the intermediary effect of interim CVD events during follow-up in the (null) association between menopausal age and AF.

## Discussion

AF affects about 1–2% of the total population. Prevalence of AF increases with advancing age, rising to around 10% in individuals older than 75 years (4, 58). Although age-adjusted AF incidence and prevalence is larger among men, women are older at the time of AF diagnosis and have higher CHA_2_DS_2_-VASc scores. Moreover, women with AF have worse quality of life and larger risk for AF-associated adverse outcomes such as morality and stroke (3, 59).

Epidemiological studies have highlighted gender differences in epidemiology of AF. Elevated BMI seems to confer a higher risk of AF among men. However, evidence regarding sex differences in the association between DM, elevated blood pressure, and dyslipidemia with AF remains conflicting. While men with AF have larger burden of coronary artery disease, women with AF tend to have a larger prevalence of heart failure and valvular heart disease.

When interpreting the results from epidemiological studies of AF, several factors need to be taken into account. Firstly, the true incidence and prevalence of AF are probably larger than those indicated by the statistics from epidemiological studies as prolonged electrocardiographic rhythm monitoring could lead to identification of clinically silent AF among subjects who present in sinus rhythm (60). Secondly, AF ascertainment in epidemiological studies is challenging and there is a large room for misclassification of AF status. The natural history of AF is complex and the exact mechanisms and factors that govern the clinical course of AF are uncertain. As AF progresses from paroxysmal to persistent and permanent forms, the prevalence of comorbidities as well as the accompanying risk factor burden might change (61). Moreover, AF may change over time as the AF-associated risk factors shift in prevalence and severity also in response to primary and secondary prevention treatments (8). These factors might affect the observed differences in epidemiology and risk factors for AF between women and men. Thirdly, observational studies may be subject to significant bias and residual confounding from incompletely measured factors. This may, in part, explain the observed differences between women and men. Moreover, as pertinent to epidemiologic research, the likelihood of false positive findings, especially in the setting of multiple comparisons, should be considered. The magnitude of the association, i.e., weak versus strong associations, should be taken into account. Epidemiological standards in the design, analysis, reporting, and interpretation of studies need to be critically assessed. Finally, characteristics of the epidemiological studies are central in interpreting the results. Therefore, understanding the differences in risk factor profile between women and men included in the studies, for example in terms of the obesity or aging profile, is essential for making solid inferences. Differences in the risk profile of women and men understudy might confound or influence interpretations.

Despite some suggestive sex-specific associations of risk factors with AF, causality remains to be stablished. Genetic studies improve our understanding of sexual dimorphism in disease burden and susceptibility to risk factors. So far, sex-specific genetic studies of AF are sparse. The recent large meta-analysis of genome-wide association (GWA) studies for AF identified 97 significant genetic loci which implicate genes enriched within cardiac developmental, electrophysiological, contractile and structural pathways (62). Sex-specific genetic associations with AF, however, were not reported in this large GWA study. Mendelian randomization approach uses genetic variants as instrumental variables to establish potential causality. This approach can be a strong alternative for better understanding the sex-specific nature of the associations of risk factors with AF. A recent large-scale Mendelian randomization study has confirmed a causal relationship between BMI and incident AF (63). However, sex differences were not reported.

Besides observations from epidemiological and clinical studies, to improve our understanding of sexual dimorphism in AF, sufficiently large hypothesis-free GWA or candidate gene studies with a priori, clearly defined hypotheses, as well as well-powered Mendelian randomization studies are needed. Possible gene-gene and gene-environment interactions should be considered. The discovered genetic loci can be taken forward for further functional studies to reveal plausible underlying sex-related pathophysiological pathways in AF. Besides investigation of plausible underlying pathophysiological mechanisms, appraisal of the suggested sex differences in the association of several risk factors with AF regarding their relevance to sex-specific prevention strategies is warranted.

## Author Contributions

MK performed the literature review and drafted the article.

### Conflict of Interest

The author declares that the research was conducted in the absence of any commercial or financial relationships that could be construed as a potential conflict of interest.
